# 284. Antibiotic Prescribing Trends in Hospitalized Influenza Versus COVID-19 Patients at a Community-Based Health System

**DOI:** 10.1093/ofid/ofab466.486

**Published:** 2021-12-04

**Authors:** Aileen Martinez, Lyssette Cardona, Nina Ricci

**Affiliations:** 1 Cleveland Clinic Martin Health, Stuart, Florida; 2 Cleveland Clinic Florida, Stuart, Florida; 3 Nova Southeastern University, Stuart, Florida

## Abstract

**Background:**

The 2019 coronavirus SARS-CoV-2 continues to affect global population health. Patients with severe disease that require hospitalization due to COVID-19 pneumonia remain at further risk of bacterial co-infections. There is limited evidence suggesting up to 3.5% bacterial co-infection upon admission and up to 13.5% of secondary infections after hospitalization for pneumonia yet antibacterial therapy usage remain as high, or even higher, than data seen for viral pneumonia, such as influenza. Unnecessary use of antimicrobial therapy may lead to further resistance and requires stewardship attention.

**Methods:**

A single-center retrospective chart review was conducted in a community health system on all inpatient influenza admissions between October 1^st^ 2019 to March 31^st^ 2020 and all COVID-19 admissions during the same 6-month period one year later. Patients were excluded if age < 18, observation or emergency visit. The study aims to determine the percentage of patients that were prescribed antibacterial therapy during influenza season compared to during the COVID-19 pandemic.

**Results:**

A total of 175 patients were included in the influenza group while 1411 patients were included in the COVID-19 group (Table 1). The percent of inpatients with positive bacterial respiratory cultures were 12% in both influenza and COVID-19 groups. Positive bacterial respiratory cultures collected within 48 hours of admission were 3.4% in the influenza group compared to 1.2% in the COVID -19 group. Seventy-three percent of patients in the influenza group received antibiotics during admission compared to 78% in the COVID -19 group. Azithromycin and/or ceftriaxone was most commonly prescribed (58% vs. 60%) (Figure 1). The median length of stay was 3 days in the influenza group compared to 5 days in the COVID-19 group. In hospital mortality was higher in the COVID-19 group (1.7% vs. 9%).

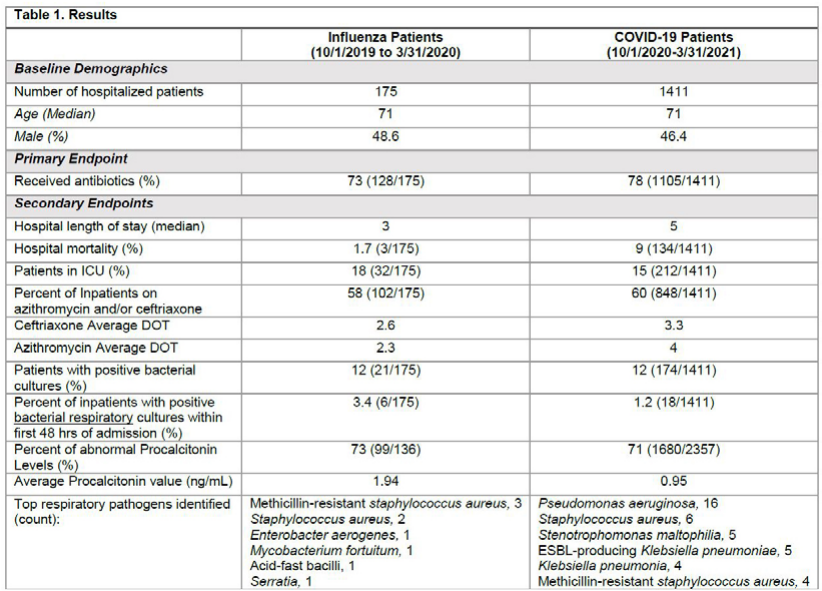

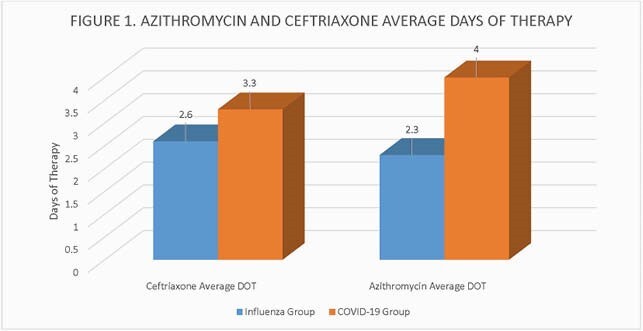

**Conclusion:**

Despite the viral origin of influenza and COVID-19 and low incidence of bacterial infection, antibacterials were frequently prescribed in both indications but it appears to trend more so in the COVID-19 group. There is an opportunity to enhance antimicrobial stewardship for the treatment of COVID-19 in acute care settings.

**Disclosures:**

**All Authors**: No reported disclosures

